# Color vision varies more among populations than among species of live-bearing fish from South America

**DOI:** 10.1186/s12862-015-0501-3

**Published:** 2015-10-16

**Authors:** Benjamin A. Sandkam, C. Megan Young, Frances Margaret Walker Breden, Godfrey R. Bourne, Felix Breden

**Affiliations:** Department of Biological Sciences, Simon Fraser University, 8888 University Drive, Burnaby, V5A 1S6 BC Canada; School of Interactive Arts and Technology, Simon Fraser University, 8888 University Drive, Burnaby, V5A 1S6 BC Canada; Department of Biology, University of Missouri-St. Louis, 1 University Blvd., 103 Research Building, St. Louis, 63121 MO USA

**Keywords:** Mate choice, Opsin, Guppy, Poeciliidae, Sensory bias, Population divergence

## Abstract

**Background:**

Sensory Bias models for the evolution of mate preference place a great emphasis on the role of sensory system variation in mate preferences. However, the extent to which sensory systems vary across- versus within-species remains largely unknown. Here we assessed whether color vision varies in natural locations where guppies (*Poecilia reticulata*) and their two closest relatives, *Poecilia parae* and *Poecilia picta*, occur in extreme sympatry and school together. All three species base mate preferences on male coloration but differ in the colors preferred.

**Results:**

Measuring opsin gene expression, we found that within sympatric locations these species have similar color vision and that color vision differed more across populations of conspecifics. In addition, all three species differ across populations in the frequency of the same opsin coding polymorphism that influences visual tuning.

**Conclusions:**

Together, this shows sensory systems vary considerably across populations and supports the possibility that sensory system variation is involved in population divergence of mate preference.

**Electronic supplementary material:**

The online version of this article (doi:10.1186/s12862-015-0501-3) contains supplementary material, which is available to authorized users.

## Background

Population divergence is widely accepted as a precursor to speciation [1], and can occur rapidly due to sexual selection via changes in mate preference [2]. Many hypotheses have attempted to explain the drivers behind changes in mate preference, which fall into two general categories— indirect models, such as Fisher’s runaway and good genes, or direct models such as Sensory Bias (reviewed in [3, 4]). Sensory Bias models emphasize the role of sensory system variation in driving divergence in mate preferences [5–9]. However, the extent to which sensory systems vary across- versus within-species remains largely unknown. Describing where the variation in sensory systems is partitioned is important for research aimed at directly testing such models of population divergence in mate choice.

**Fig. 1:**
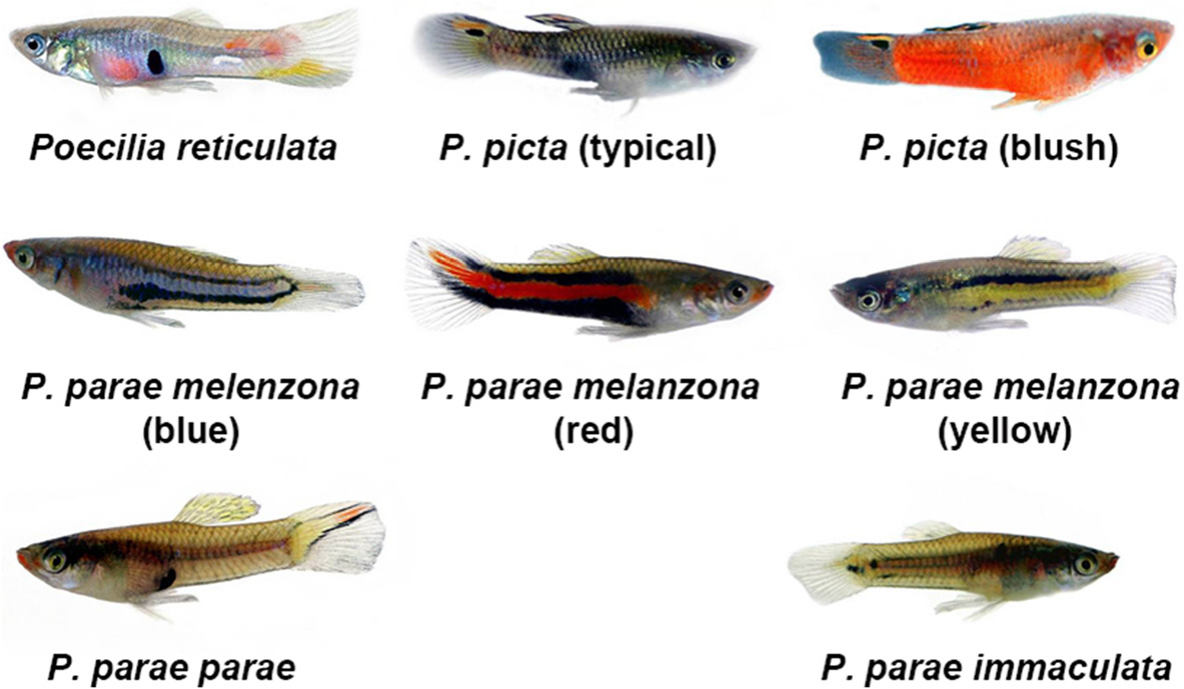
Male morphs of the three sympatric species

Guppies have been a valuable model for studies of the evolution of female mate preferences based on visual signals for nearly 100 years (reviewed in [10, 11]). Recently, *P. reticulata* has been shown to vary in the tuning of color vision across populations in a manner that correlates with female mate preferences on the island of Trinidad [12]. In contrast to Trinidad, populations of guppies from mainland South America frequently occur in extreme sympatry and commonly school with two of their closest relatives, *Poecilia picta* and *P. parae* [13–14]. These species occupy similar ecological niches and have highly similar morphometrics, with the largest differences between species being male coloration [13, 15–17]. In all three species, female mate preferences largely rely on male visual cues, yet the male traits preferred by females differ across species [13, 15, 18, 19]. Males of *P. reticulata* have highly variable numbers and colors of spots on their body [13, 20, 21]. In *P. parae*, males occur in one of five discreet Y-linked morphs: three uni-color morphs (with color pattern dominated by a horizontal stripe that is either: red, yellow, or blue), one female mimic morph (colored like the female and engaging in sneak copulations), and one large, aggressive morph (with vertical dark bars and most of its coloration on the caudal fin) [15, 22]. Males of *P. picta* all have an orange stripe on their caudal fin and yellow bands on their dorsal fin [13]. While most males of *P. picta* have no additional color, some males occur as a morph with red running diffusely throughout the entire body, although this red coloration doesn’t strongly influence mating [17] (see Fig. 1 for pictures of the male morphs of all three species). While males occasionally perform courtship displays for heterospecific females, female *P. picta* do not accept heterospecific males as mates [13, 16, 23]. The variable role of color in female mate choice [24, 25], high similarity in niche and morphology, and occurrence in sympatry make these species, *P. reticulata*, *P. picta*, *P. parae*, an excellent system with which to examine whether color vision varies more across species or populations.

Color vision is accomplished by comparing the signals from different cone cells in the retina, which are maximally tuned to different wavelengths of light [26]. The wavelength at which a cone cell maximally detects light is primarily determined by the transmembrane protein expressed, called an opsin [27, 28]. *P. reticulata*, *P. picta*, and *P. parae* have an astounding nine cone opsin proteins, among the highest for vertebrates [29–31]. Each cone opsin is coded by a single gene which is grouped and named for the range of light they detect: SWS1 (SWS1, short wavelength-sensitive) detects ultra-violet; SWS2A and SWS2B (SWS2, short wavelength sensitive 2) detect blues and purples; RH2-1 and RH2-2 (RH2, rhodopsin-like) detect greens; and LWS-1, LWS-2, LWS-3, and LWS-R (LWS, long wavelength-sensitive) detect reds and oranges [30–33].

Differences in tuning of color vision can occur through changes in either gene sequence or expression (reviewed in [34]). Guppies have been shown to vary in tuning of color vision across populations through both differences in the frequency of an allele known to affect tuning of LWS-1 [12, 33] and also differences in opsin expression profiles [12]. Opsin expression profiles provide an estimate of cone cell proportions in the retina and thereby offer an excellent measure of the allocation of an individual’s color vision repertoire to different cone cell types [12, 35–40]. Guppy populations with stronger female preferences for males with more red/orange coloration have higher expression of LWS opsins [12].

Here, we examined whether there is more variation in visual tuning within species or across species. The occurrence of multiple sympatric locations of the three closely related species *P. parae*, *P. picta*, and *P. reticulata* on mainland South America allows us to examine differences in visual tuning of all three species from the same environment in a replicated manner across populations. By examining visual tuning of each species across multiple locations we are able to assess the variability of opsin expression across populations and compare this to species differences.

## Methods

### Sample collection

We sampled *Poecilia parae, P. picta,* and *P. reticulata* from four sympatric locations within Guyana between 10:00 and 16:00 in June-July 2010 (see Additional file 1 for map and Additional file 2: Table S1 of GPS coordinates). One sympatric location (Seawall Trench) was sampled on two days. Efforts were made to collect five adult males and five adult females of each species at every location; however not all species were present at the same density within locations resulting in smaller sample sizes for some collections (see Additional file 2: Table S1 of sample sizes of each species and population). *P. bifurca* is a close relative of *P. reticulata*, *P. picta*, and *P. parae* but does not occur sympatrically. Opsin expression for one population of *P. bifurca* (~79 km from closest sympatric location sampled) is reported here only as a qualitative comparison and is not included in statistical analyses. All four species occur in similar environments; small drainage ditches of Guyana that are usually only a few meters wide and less than a meter deep [13]. Adult males show pronounced species specific coloration, while females are all grey with minor differences in black patterning around the urogenital opening; these traits allow us to rapidly perform visual identification of species and sex [41]. Sampling followed the protocols of Sandkam et al. [12]. Briefly, adult fish were caught with dip nets— individuals were rapidly sacrificed in an overdose of MS-222, measured and photographed. We immediately removed eyes and made a small puncture to facilitate complete penetration of RNA*later®* Stabilization Solution (Life Technologies™). Both eyes from an individual were placed into a vial of RNA*later*® and kept on ice for 24 h, to allow tissue to be saturated per manufacturer’s recommendation. After 24 h, we transferred the vials to liquid nitrogen. The vials were removed from liquid nitrogen just prior to being placed in checked baggage and flown to Simon Fraser University where we placed them in a −20 °C freezer until RNA extraction. Time spent at room temperature totaled less than 24 h and fell well under the one-week maximum suggested by manufacturer. The bodies of individuals sampled were placed in tubes of 95 % EtOH buffered with EDTA and kept at −20 °C until DNA extraction.

### qPCR assay design

To measure opsin expression, we designed qPCR assays following the methods of Sandkam et al. [12]. We modified the primers reported in Sandkam et al. for *P. reticulata*, such that one set of qPCR assays could be used across all four species. Sequences from *P. reticulata*, *P. picta*, *P. parae*, and *P. bifurca* were aligned and viewed using SeqMan Pro (Lasergene 8.0; DNASTAR, Madison, WI). We designed probe based PrimeTime® qPCR assays (IDT® Technologies) in regions of conserved sequence such that there were no SNPs between any of the four species for all of the primer/probe assays. Assays were designed to be specific for each of the nine opsins, one rhodopsin (RH1), and three housekeeping genes (beta actin (B-actin); cytochrome c oxidase subunit I (COI); myosin heavy chain (Myosin HC)). Whenever possible, primers spanned intron-exon boundaries. Each assay consisted of a forward primer, reverse primer and 5’ FAM labeled probe with both 3’ Iowa Black® and internal ZEN™ quenchers (IDT® Technologies) (see Additional file 2: Table S2 of primer/probe sequences and product length). Assay specificity for each species was verified by the presence of a single band when running PCR products on an agarose gel. Within each species, LWS-1 and LWS-R assays resulted in products of the same size, while LWS-1 and LWS-3 loci are similar in sequence. To ensure that LWS-1, LWS-3, and LWS-R were truly locus specific assays, we measured the pairwise covariance of these three assays on the final relative_(hk)_ data set (described below) using R v3.0.2. If assays were binding to non-specific targets we would expect to see large positive covariances between assays. We found no substantial covariance in any of the four species between either LWS-1 and LWS-R (covariance: *P. reticulata*, −9*10^−6^; *P. parae*, 0.0011; *P. picta*, −0.001; *P. bifurca*, −0.0028) or LWS-1 and LWS-3 (covariance: *P. reticulata*, −1*10^−6^; *P. parae*, 0.0066; *P. picta*, 0.0004; *P. bifurca*, 0.0059), demonstrating locus specificity of the LWS assays.

We determined the relative PCR efficiency (*E*_*i*_) for each assay as in Sandkam et al. [12], using four gBlocks® Gene Fragments (synthetic double stranded, sequence-verified genomic blocks made by IDT® Technologies). gBlocks® were designed on sequence from *P. reticulata* and contained sequence for each of the genes being assayed from 20 bp upstream of the forward primer to 20 bp downstream of the reverse primer. To ensure equal proportions of each gene when calculating relative efficiency, we adjusted the length of each opsin gBlocks® to 728 bp by adding upstream and downstream sequence from the first and last opsin. There were less than seven SNPs per gene across the four species in regions spanned by the assays and none of these differences occurred in primer/probe sites. Relative primer efficiencies calculated using these constructs were used for all four species. Gene order in the gBlocks® was randomized: gBlock® 1 contained LWS-1, RH1, SWS1, LWS-R; gBlock® 2 contained SWS2B, LWS-3, RH2-2; gBlock® 3 contained LWS-2, SWS2A, RH2-1; and gBlock® 4 contained B-actin, COI, Myosin-HC. The 4 gBlocks® were mixed in equal proportions and brought to a concentration of 0.001 ng/μl resulting in a control with equal ratios of all the opsin and housekeeping genes. We ran six replicates of each assay using 4.5 μl of the control. The relative primer efficiencies (*E*_i_) were then calculated following Carleton and Kocher [42] using the equation:

$$ \frac{{\left(1+1\right)}^{C{t}_{High}}}{{\left(1+{E}_i\right)}^{C{t}_i}}=1 $$

such that *Ct*_High_ is the critical threshold of the opsin with the highest expression (lowest *Ct* value) and *Ct*_i_ is the critical threshold for opsin *i*. The mean relative efficiency and standard error was calculated across the six replicates (see Additional file 2: Table S2 of assay efficiencies).

The SWS2A assay had the highest relative efficiency and was used to measure absolute efficiency. A thousand fold serial dilution was made of a random sample of *P. picta*. Three replicates of qPCR were performed on each concentration using the SWS2A assay. The absolute efficiency of SWS2A was found using the slope of *ln* (concentration) plotted on *Ct* such that *E*_*SWS*2*A*_ = *e*^− *slope*^ − 1. The absolute efficiencies of the other primer/probes were determined following Fuller et al. [43] using the equation:

$$ \mathrm{absolute}\ {E}_i=\left(\mathrm{relative}\ {E}_i\times \mathrm{absolute}\ {E}_{SWS2A}\right)/\mathrm{relative}\ {E}_{SWS2A} $$

### Sample processing and analyses for opsin expression

Sample processing followed Sandkam et al. [12]. We placed both eyes from one individual in 600 uL of TRIzol® reagent (Life Technologies™) and ground them with a 1.5 mL RNase-free Kontes® Pellet Pestle Grinder (Kimble Chase). Solution was then run through an Ambion® Homogenizer (Life Technologies™) to reduce viscosity. We extracted RNA following the manufacturer’s instructions using PureLink® RNA Mini Kits with the addition of on column treatments with PureLink® DNase (Life Technologies™) to eliminate any potential genomic contamination during qPCR. To verify quality of extracted RNA we ran a subset of samples on an Experion Bioanalyzer. RNA concentrations were adjusted to 50 ng/uL using UltraPure™ DNase/RNase-Free Distilled Water (Life Technologies™). For each sample 500 ng RNA was reverse transcribed using a High Capacity cDNA Reverse Transcription Kit with RNase Inhibitor (Life Technologies™) following manufacturer’s instructions. cDNA samples were diluted roughly 20-fold using UltraPure™ DNase/RNase-Free Distilled Water (Life Technologies™) for use in qPCR reactions. Triplicate qPCR reactions were run on each individual for the nine opsins, one rhodopsin, and three housekeeping genes using the qPCR probe based assays described above. We ran all 39 reactions for each individual simultaneously on the same 384 well plate in addition to negative controls (UltraPure™ water) for each assay. Each 10 uL reaction consisted of: 5 uL Brilliant III Ultra-Fast qPCR Master Mix (Agilent Technologies), 0.5 uL FAM labeled assay (described above) and 4.5 uL sample. We set up all reactions on ice and the plates were briefly spun down before being run on an Applied Biosystems® 7900HT qPCR machine (Life Technologies™). PCR conditions were as follows: 95 °C for 3:00 followed by 40 cycles of 95 °C for 0:05, 60 °C for 0:15. The standard deviation of the triplicate reactions was taken and when >2, outliers were removed (comprising only 5 % of the 5538 reactions for this study).

We assessed differences in color vision by calculating the proportion of total opsin expression (*T*_all_) made up of each opsin (*T*_i_) following Fuller et al. [43] and Carleton and Kocher [42] with the following equation:

$$ \frac{T_i}{T_{all}}=\frac{\left(1/\left({\left(1+{E}_i\right)}^{C{t}_i}\right)\right)}{{\displaystyle \sum \left(1/\left({\left(1+{E}_i\right)}^{C{t}_i}\right)\right)}} $$

where *E*_*i*_ is the mean primer/probe efficiency of assay *i* and *Ct*_*i*_ is the mean critical cycle number for gene *i* (expression of the nine opsins adds to one for each individual).

We assessed differences in regulation of the nine cone opsins and one rhodopsin genes by comparing expression relative to housekeeping genes (*T*_*House*_). To control for random variation in housekeeping gene expression, we took the average of three housekeeping genes [44]. We calculated measures following Sandkam et al. [12] using the equation:

$$ \frac{T_i}{T_{\mathrm{House}}}-=\frac{\left(1/{\left(1+{E}_i\right)}^{C{t}_i}\right)}{\left(\left({\displaystyle \sum \left(1/{\left(1+{E}_{House}\right)}^{C{t}_{House}}\right)}\right)/3\right)} $$

where *E*_*House*_ is the primer/probe efficiency for a housekeeping gene and *Ct*_*House*_ is the critical cycle number for that gene.

This resulted in each individual having two measures for each opsin: the proportion of total opsin expression made up by that opsin (proportional), and relative to housekeeping genes (relative_(hk)_). As opsins are the major differentiating character of cone cell types, and color vision is accomplished by comparing the signal from different cone cell types, proportional measures of opsin expression provide a measure of color vision [43, 45]. Differences in regulation of individual opsins compared to overall gene transcription are revealed by relative_(hk)_ measures of opsin expression, whereas overall gene activity is measured as the mean of the three housekeeping genes [12, 45].

### LWS-1 A/S allele frequency

Variation in visual systems across species or populations can also occur through differences in the frequency of polymorphisms known to alter tuning of the visual pigments. Only LWS-1 is known to possess a polymorphism that alters spectral tuning in *P. parae*, *P. picta*, or *P. reticulata* [12, 30, 33]. The key difference between these alleles is the presence of either a serine or alanine as the amino acid at the position that corresponds to residue 180 in human M/LWS opsins (termed ‘LWS-1 (180 Ser)’ and ‘LWS-1 (180 Ala)’ respectively). This polymorphism can result in a change of tuning of the LWS-1 opsin protein by up to 7 nm [30, 33]. To determine species and population frequencies of the LWS-1 (180 Ala) and LWS-1 (180 Ser) polymorphism we extracted genomic DNA from tail tissue of the same individuals we used for expression analyses using a DNeasy blood and tissue kit (Qiagen). We generated PCR products using 5’ and 3’ UTR-specific primers of the LWS-1 locus. Internal sequencing primers were used directly on PCR products to generate chromatograms, spanning part of exon 2 and all of exon 3, at Molecular Cloning Laboratories (McLab, San Francisco, CA) (see Additional file 2: Table S3 of primer sequences). We viewed and analyzed the sequencing chromatograms using SeqMan Pro (LASERGENE 8.0; DNASTAR, Madison, WI). For each individual, we determined if the 180 amino acid residue was either a serine, alanine or heterozygous. We calculated genotype and allele frequencies for each species in each location. *F*_*ST*_ values based on the frequency of alleles with a serine at amino acid position 180 were calculated two ways; between species within the same location, and within species across populations. *F*_*ST*_ values were calculated as in Sandkam et al. [12], using:

$$ {F}_{ST}=\frac{\operatorname{var}(S)}{\overline{S}\times \left(1-\overline{S}\right)} $$

where var(*S*) is the variance of the frequency of the serine allele, either across species (within locations) or across populations (within species), and $\overline{S}$ is the frequency of the serine allele in either the location or species (respectively).

### Statistical analyses of opsin expression

#### Do species and/or populations differ in color vision?

To test whether closely related sympatric species differ in either proportional or relative_(hk)_ measures of opsin expression profiles, we ran MANOVAs with the levels for each of the nine cone opsins log transformed and treated as dependent variables. Sex was nested within Species and since Locations were sampled at different times of the day, which can impact opsin expression [12], we further nested Species within Location. All expression data were analyzed using R v3.0.2 [46].

Sex had no main or interaction effect (Table 1). There are no differences between sexes in opsin expression of natural guppy populations on Trinidad [12], nor for opsin expression in most other species in which it has been examined (such as stickleback (*Gasterosteus aculeatus*) [47], and killifish (*Lucania goodei*) [48]). Therefore we pooled sexes for all of the following analyses.

**Table 1:** Results of MANOVA on opsin expression profiles as proportional and relative_(hk)_ measures

		Proportional	Relative_(hk)_
*Loc*	*F*_(3,109)_	**7.13**	**5.87**
	*P*	**<0.0001**	**<0.0001**
*Sp(Loc)*	*F*_(8,109)_	**1.61**	**2.04**
	*P*	**0.0014**	**<0.0001**
*Sx(Sp(Loc))*	*F*_(12,109)_	1.13	0.98
	*P*	0.1872	0.5490

*Loc*, Location; *Sp(Loc)*, Species nested in Location; *Sx(Sp(Loc))*, Sex nested in Species, nested in Location. Bold indicates *P* < 0.05

#### Does opsin expression differ more by species or locations?

Akaike Information Criterion (AIC) scores were calculated by Species, Location, or a Species*Location interaction to determine the ability of models built on these factors to explain variation in proportional expression for each of the opsin genes.

#### How do species differ in color vision?

To better understand differences in color vision across species within the same location, we ran independent ANOVAs by Species on data subset by Location. Opsins that significantly differed in ANOVAs were followed up with a Tukey test to determine which of the species contributed to the significant effects.

## Results

### Do species and/or populations differ in color vision?

**Fig. 2:**
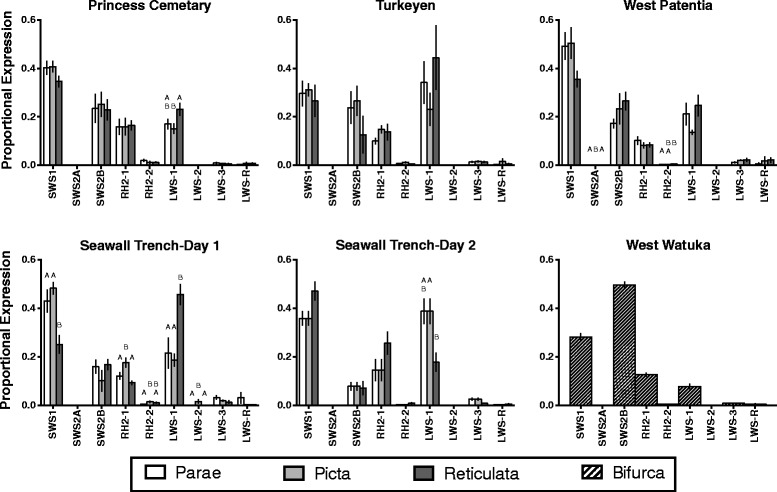
Proportional measures of opsin expression across species and locations. Letters denote significant differences in expression of individual opsins across species within a location. Note: West Watuka has only *P. bifurca* and was not used in statistical analyses

Across all locations and species SWS2A, RH2-2, LWS-2, LWS-3, and LWS-R showed rather low levels of expression compared to other opsin genes (Figs. 1, 2). The most abundant opsin expressed was either SWS1 or LWS-1 but varied across locations and species (Fig. 2). MANOVAs revealed that color vision, as measured by differences in opsin expression, varied significantly within species across populations and within locations across species (Table 1). However, expression profiles did not vary by sex (nested in species-in locations) for either proportional (*F*_12,109_ = 1.1275, *P* = 0.1872) or relative_(hk)_ (*F*_12,109_ = 0.977, *P* = 0.5490) measures.

### Does opsin expression differ more by species or location?

We found opsin expression to vary significantly by Location and Species nested within Location. To determine if Species, Location or a Location*Species interaction best explained variation in proportional expression we calculated AIC scores. Location alone best explained variation in SWS1, SWS2B, RH2-1, RH2-2, and LWS-1 (Table 2). Location and Location*Species explained the variation equally well for SWS2A and LWS-3, as seen by AIC scores differing by less than 2 for these two models in both genes. Location and Species explained variation of LWS-R equally well with AIC scores within 1 for these two models. Only for LWS-2 did Species best explain variation in opsin expression (Table 2). Overall, we found that variation in color vision, as measured by opsin expression, was better explained by Location than by Species.

**Table 2:** Result of AIC analyses

	df	SWS1	SWS2A	SWS2B	RH2-1	RH2-2	LWS-1	LWS-2	LWS-3	LWS-R
Location*Species	13	−156.12	**−2015.89**	−147.09	−244.66	−847.86	−71.63	−1022.10	**−678.85**	−626.69
Location	5	**−160.52**	**−2014.14**	**−154.32**	**−255.37**	**−855.18**	**−78.62**	−1024.31	**−677.00**	**−630.76**
Species	4	−152.08	−2008.59	−134.21	−250.90	−839.24	−70.27	**−1027.32**	−669.96	**−631.74**

AIC values for the ability of models based on Location, Species, or Location*Species to explain variation in proportional measures of opsin expression for each opsin gene. Bold indicates model with lowest AIC value for each opsin

### How do species differ in color vision?

**Fig. 3:**
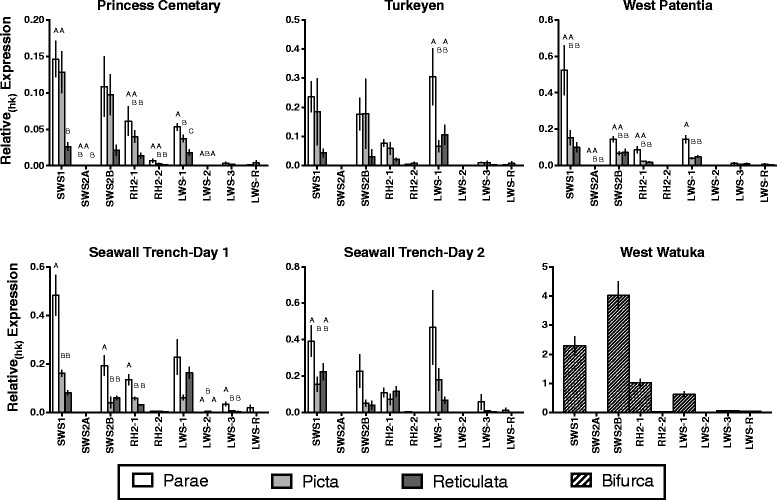
Relative_(hk)_ measures of opsin expression across species and locations. Letters denote significant differences in expression of individual opsins across species within a location. Note: West Watuka has only *P. bifurca* and was not used in statistical analyses

When relative_(hk)_ measures of opsin expression differed by species, *P. parae* generally had highest expression (Fig. 3). Despite differences in the abundance of opsin expression, color vision was highly similar across species, as indicated by few occurrences in which locations differed in proportional measures of opsin expression across species (10 of 45 possible opsin by location combinations tested) (Table 3).

**Table 3:** Results of opsin expression ANOVAs using proportional and relative_(hk)_ measures by location

		Princess Cemetery		Seawall Trench				Turkeyen		West Patentia	
				Day 1		Day 2					
		*F*_(2,27)_	*P*	*F*_(2,21)_	*P*	*F*_(2,28)_	*P*	*F*_(2,23)_	*P*	*F*_(2,19)_	*P*
Proportional Measures	SWS1	1.97	0.1590	**10.11**	**0.0008**	2.15	0.1350	0.21	0.8100	3.03	0.0722
	SWS2A	3.22	0.0556	2.64	0.0951	0.25	0.7810	0.83	0.4500	**23.86**	**<0.0001**
	SWS2B	0.06	0.9460	1.37	0.2750	1.24	0.3060	0.98	0.3911	2.40	0.1170
	RH2-1	0.02	0.9840	**9.94**	**0.0009**	2.85	0.0747	2.09	0.1470	0.28	0.7590
	RH2-2	0.94	0.4030	**4.44**	**0.0247**	0.79	0.4660	1.89	0.1730	**8.56**	**0.0022**
	LWS-1	**3.45**	**0.0464**	**10.03**	**0.0009**	**5.17**	**0.0123**	1.26	0.3020	0.62	0.5470
	LWS-2	1.71	0.1990	**4.88**	**0.0182**	0.86	0.4350	1.20	0.3200	**4.01**	**0.0354**
	LWS-3	0.38	0.6880	2.58	0.0999	2.41	0.1090	0.12	0.8880	0.57	0.5740
	LWS-R	0.60	0.5580	1.65	0.2160	0.96	0.3960	1.03	0.3730	1.59	0.2290
Relative_(hk)_ Measures	SWS1	**8.84**	**0.0011**	**19.99**	**<0.0001**	**4.35**	**0.0227**	1.14	0.3370	**5.30**	**0.0149**
	SWS2A	**4.22**	**0.0253**	0.90	0.4220	0.76	0.4790	0.75	0.4820	**4.47**	**0.0256**
	SWS2B	2.71	0.0847	**9.98**	**0.0009**	**3.85**	**0.0332**	0.73	0.4920	**5.55**	**0.0126**
	RH2-1	**3.46**	**0.0429**	**18.74**	**<0.0001**	0.76	0.4760	2.65	0.0922	**7.49**	**0.0040**
	RH2-2	**4.19**	**0.0259**	2.04	0.1550	1.52	0.2370	0.80	0.4630	0.82	0.4550
	LWS-1	**15.04**	**<0.0001**	2.94	0.0747	2.91	0.0711	**3.99**	**0.0323**	**13.52**	**0.0002**
	LWS-2	**10.05**	**0.0005**	**5.67**	**0.0108**	0.16	0.8560	0.56	0.5800	1.32	0.2030
	LWS-3	1.75	0.1930	**11.42**	**0.0004**	1.65	0.2110	0.68	0.5160	0.44	0.6520
	LWS-R	1.22	0.3110	2.34	0.1210	1.15	0.3310	1.00	0.3820	0.72	0.5000
	RH1	**5.55**	**0.0096**	**5.73**	**0.0103**	2.66	0.0876	**6.19**	**0.0045**	**12.64**	**0.0003**

Bold indicates *P* < 0.05

#### LWS-1 A/S allele frequency

**Fig. 4:**
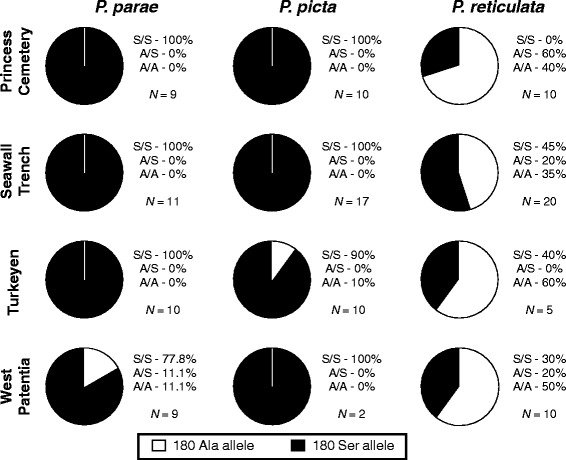
Gene frequencies. Genotype and gene frequencies of the 180 Ala (A) vs. 180 Ser (S) allele of LWS (long-wavelength sensitive)-1 gene

We found within-species gene frequencies were similar across locations, but across-species gene frequencies differed within locations such that *P. reticulata* showed higher frequencies of the LWS-1 (180 Ala) allele than either *P. parae* or *P. picta* (Fig. 4). The *F*_ST_ scores we observed confirmed low differences within species in the frequency of the serine allele across locations, with the most variation occurring in *P. parae* (Table 4). The within location differences in frequency of the serine allele observed across species were moderate (Table 5). This is a result of the high frequency of LWS-1 (180 Ala) in *P. reticulata* while it is nearly absent in *P. parae* and *P. picta*. However, while qualitative differences in frequency and *F*_ST_ scores are clear, one should take care when interpreting our results quantitatively as the samples sizes were low (ranging from 2–20 individuals) (Fig. 4).

**Table 4:** *F*_st_ based on the frequency of the 180 Ser allele of LWS-1. Calculated across populations of the same species

Within Species – Across Populations			
	*P. parae*	*P. picta*	*P. reticulata*
*F*_st_	0.130	0.077	0.033

**Table 5:** *F*_st_ based on the frequency of the 180 Ser allele of LWS-1. Calculated across species in the same location

Within Locations – Across Species				
	Princess Cemetery	Seawall Trench	Turkeyen	West Patentia
*F*_st_	0.609	0.353	0.385	0.336

## Discussion

Intersexual mate choice was first proposed as a mechanism for speciation through divergence in the traits preferred [49]. Over time, divergence of populations has widely been accepted as the first step toward speciation [1]. Therefore, understanding the factors involved in population divergence of mate choice has far reaching implications to many fields including animal behavior and evolutionary biology. Sensory Bias models for the evolution of mate preference place a strong emphasis on the role of sensory system variation in mate preferences [5–9]. However, the extent to which sensory systems vary across, versus within, species remains largely unknown. Here we describe variation in color vision across three closely related sympatrically occurring species. We found that color vision varies across populations but does not vary consistently between species across locations. Below we describe the variation we found and discuss our findings in the framework of mate choice evolution.

### Color vision differs across populations within species

Visual systems are classically thought to show low to no variation across individuals within a species, especially when color vision is modeled in the context of mate choice (such as [19, 50–52]). Yet color vision can vary through differences in the abundance of different cone cell types, amino acid sequences of the opsin proteins, or neural processing of signals from the eyes [34]. Opsin expression profiles provide an estimate of the ratio of cone cells expressing different opsins in the eyes, which can impact color vision [12, 34, 42, 43]. We present the first study of opsin expression in *P. bifurca*, *P. parae* and *P. picta*, and one of the few to carefully compare within versus between species color vision in similar environments. We demonstrate that color vision in these species, and sympatric *P. reticulata*, likely differs across populations through differences in opsin expression, and could differ through frequency of an alternative amino acid sequence.

Color vision of *P. parae* from nearby Guyanan populations has recently been explored using microspectrophotometry (MSP) to identify the peak wavelength sensitivity (λ_max_) of the cone cells [19]. While *P. parae* possess nine opsin genes, Hurtado-Gonzales and colleagues only found seven cone cell types across individuals. This can be explained by the low-to-no expression of both SWS2A and LWS-2 we found, emphasizing the important role of expression in tuning the visual system. While our measures of opsin gene expression provide insight to differences in color vision, it is possible that the exact cone cell proportions could differ from measures of proportional RNA expression through differences in non-transcriptional control such as translation rates.

The long wavelength-sensitive-1 (LWS-1) opsin is the only opsin in Poeciliidae known to vary in tuning due to differences in amino acid sequence. The LWS-1 opsin can either have an Alanine or Serine at the site corresponding to the 180^th^ amino acid in the human opsin sequence and is therefore termed: LWS-1 (180 Ala) and LWS-1 (180 Ser) respectively. The frequency of these two alleles varies across populations in *P. reticulata* [12,33]. This same variation is also thought to be the only sequence difference in important tuning sites across the species *P. reticulata, P. parae*, *P. picta*, and *P. bifurca* where as the later three have, until now, been thought to be fixed for either LWS-1 (180 Ala) or (180 Ser) [30]. Using the ‘five site rule’ [53], Tezuka et al. [33] estimated the λ_max_ of the LWS-1 (180 Ala) to be ~553 nm and LWS-1 (180 Ser) to be ~560 nm. Previously *P. parae* was thought to possess only one LWS-1 allele (180 Ser), however we show that *P. parae* also possesses the alternate LWS-1 (180 Ala), albeit at a low frequency. Interestingly, only 5 out of the 17 individuals (29.4 %) examined by Hurtado-Gonzales et al. [19] possessed cone cells with a λ_max_ of 553 ± 1.9 nm, this matches the predicted λ_max_ of LWS-1 (180 Ala) [33]. The closest location to the populations sampled by Hurtado-Gonzales et al. we sampled for this study was West Patentia, where 22.2 % of individuals had at least one copy of LWS-1 (180 Ala). The close proximity and lack of geographic barrier between these two populations makes it likely that allele frequencies are similar in these populations. The close frequencies of individuals with λ_max_ of 553 nm [19] and those with the LWS-1 (180 Ala) (this study) suggest variation in allele frequency across populations likely explains the presence/absence of cone cell types across individuals and further demonstrates that color vision varies across individuals in *P. parae*. We found differences across populations in the frequency of LWS-1 (180 Ala) in all three species (except for the allopatric species, *P. bifurca* which appears fixed for LWS-1 (180 Ala)) and demonstrate that color vision differs across populations not only in opsin expression, but also through differences in tuning of the opsins.

The frequency of the LWS-1 (180 Ala) allele varies across *P. reticulata* populations in Trinidad such that low predation populations are nearly fixed for the LWS-1 (180 Ser) allele while high predation populations had a greater frequency of the LWS-1 (180 Ala) allele. All the populations we use in this study are in the lowland region of Guyana where predation is extremely high (predators include birds, snakes, caiman and many fishes). Interestingly, for all of our populations of *P. reticulata*, the frequency of the LWS-1 (Ala) allele was as high or higher than the high predation populations of Trinidad [12] highlighting the potential role of predation in determining LWS-1 allele frequencies.

We also found the closely related but not sympatric species, *P. bifurca,* has a similar color vision system to *P. picta*, *P. parae* and *P. reticulata. P. bifurca* lives in environments that are generally tannin stained and occur further inland, thereby further demonstrating the surprisingly conserved nature of color vision across species in this group.

It should be noted that sites were not all sampled at the same time of day, therefore differences across populations could be confounded with diurnal variation in opsin expression, which has been shown to occur in Trinidadian guppy populations [12]. However, the diurnal differences across locations are expected to occur in all three of the sympatric species; therefore we feel our cross- versus within-species comparisons are justified.

### Sensory variation and mate choice divergence

For sensory systems to play a role in mate choice divergence there needs to be variation in sensory systems [9, 54]. We recently found support for the Sensory Exploitation model to explain population divergence in female mate preferences of Trinidadian guppies (*Poecilia reticulata*) [12] such that populations with stronger female preferences for orange male coloration expressed higher levels of LWS opsins. These results raised two important questions regarding the feasibility of sensory variation to explain mate choice differences: (1) was population variation in sensory systems present before the split of the guppy from the clade of *P. picta* and *P. parae*, and (2) if there is variation in other species, do visual systems vary more across species or populations?

We found visual systems do vary in both opsin gene expression and LWS-1 allele frequency across populations of *P. parae*, *P. picta*, and *P. reticulata* on mainland South America. Although these three species occur in such extreme sympatry that they frequently school together, they exhibit female preferences for males with different color traits [13, 18, 55]. All three of these species are known to vary in the frequency of their respective different male morphs across populations (*P. parae* [19], *P. picta* [17], *P. reticulata* [56]). It will be especially interesting for future work to determine if the variation we found in visual systems across populations correlates with differences in mate choice.

As more species are shown to vary in sensory systems across populations (e.g. Bluefin killifish [43], cichlids [57], stickleback [58], sand goby [59], pied flycatchers [60], guppies [12]), Sensory Bias models become a more likely candidate to explain divergence in mate preferences across species. This raises the question of whether differences in mate preference across species are maintained through consistent differences in their peripheral sensory systems. We found variation in color vision, as measured by opsin expression, is better explained by location than by species for most of the opsins, showing no consistent differences across species. Our data suggest mate preferences are likely maintained at another level of preference (such as higher order processing). However, we did find that the frequency of the LWS-1 (180 Ala) allele not only differs across populations, but also across species. This raises the possibility that the minor tuning differences between the LWS-1 (180 Ala) and LWS-1 (180 Ser) alleles could also play a role in mate choice differences across species. It will be interesting for future work in these species to investigate the correlation between color preference and genotype to further our understanding of visual tuning and mate preference.

## Conclusion

We show that color vision, as measured by opsin expression, differs more across populations of the same species than across species in the same location. These differences provide support for Sensory Bias models to explain population divergence in mate preference since these models rely on sensory system differences across populations. Opsin expression did not differ consistently between species across locations, suggesting species level differences in mate preference are likely maintained at levels of higher order processing.

## Ethics Statement

All work was approved by Simon Fraser University Animal Care under protocol 982B-06 and the Environmental Protection Agency of Guyana under permit # 120710 BR 135.

## Availability of Supporting Data

- All expression data and R files with statistical analyses are available on the Dryad Digital Repository: http://dx.doi.org/10.5061/dryad.s0054.
- LWS-1 sequences are available on GenBank (accession numbers: KT905869 - KT905998).

## Additional files

Additional file 1: Figure S1. Map of field sites in Guyana. (Available under a Creative Commons license). (PDF 1766 kb) Additional file 2: Table S1. Supplementary Tables. (PDF 60 kb)

## Abbreviations

λ_max_: Peak Wavelength sensitivity of a cone cell
AIC: Akaike information criterion
B-actin: Beta actin
COI: Cytochrome C oxidase subunit I
LWS: Long wavelength sensitive
MSP: Microspectrophotometry
Myosin HC: Myosin heavy chain
RH2: Rhodopsin-like
SWS: Short wavelength sensitive
