# Supplementary material for: Color vision varies more among populations than among species of live-bearing fish from South America
Source: BMC Evol Biol. 2015 Oct 16;15:225. doi: 10.1186/s12862-015-0501-3 (PMC4609137; doi:10.1186/s12862-015-0501-3)
Supplement: Additional file 1: Figure S1. — Map of field sites in Guyana. (Available under a Creative Commons license). (PDF 1766 kb) [file 12862_2015_501_MOESM1_ESM.pdf]

Field Sites

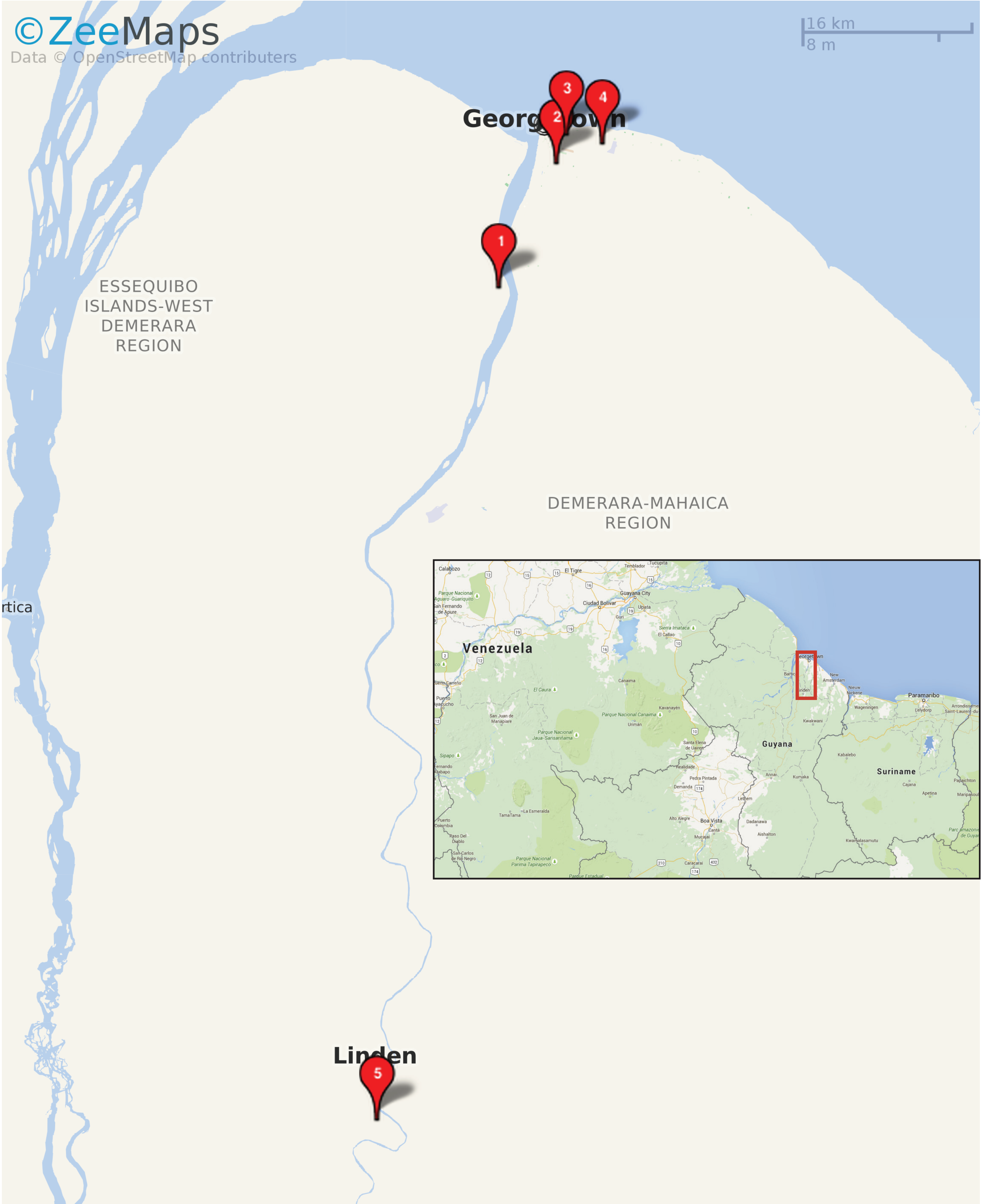

1. Patentia 2. Princess Cemetery 3. Seawall Trench 4. Turkeyen 5. West Watuka (Poecilia bifurca)
