# Supplementary material for: Color vision varies more among populations than among species of live-bearing fish from South America
Source: BMC Evol Biol. 2015 Oct 16;15:225. doi: 10.1186/s12862-015-0501-3 (PMC4609137; doi:10.1186/s12862-015-0501-3)
Supplement: Additional file 2: Table S1. — Supplementary Tables. (PDF 60 kb) [file 12862_2015_501_MOESM2_ESM.pdf]

Supplementary Table S1. GPS location and sample sizes for each location. Note: West Watuka has only *P. bifurca* and was not used in statistical analyses.

	GPS-N	GPS-W	Date	Time Sampled	Salinity (d20/20)	*P. parae*		*P. picta*		*P. reticulata*		*P. bifurca*	
						M	F	M	F	M	F	M	F
Princess Cemetery	06º 48.045’	058º 09.086’	7/14/10	10:00	1.002	5	5	5	5	5	5	-	-
Seawall Trench – Day1	06º 49.520’	058º 08.637’	7/12/10	15:30	1.003	5	3	5	1	5	5	-	-
Seawall Trench – Day2	06º 49.520’	058º 08.637’	7/13/10	10:00	1.002	5	5	6	5	5	5	-	-
Turkeyen	06º 49.067’	058º 06.764’	7/8/10	13:00	1.000	5	5	5	5	4	2	-	-
West Patentia	06º 41.752’	058º 12.066’	7/11/10	12:00	1.003	5	5	1	1	4	6	-	-
West Watuka	05º 59.509’	058º 18.282’	7/9/10	12:30	1.000	-	-	-	-	-	-	4	5

Supplementary Table S2. Relative and absolute efficiency (% efficiency) of qPCR assays.

	Forward Primer	Probe	Reverse Primer	Product Length	Relative Efficiency	Standard Error	Absolute Efficiency
B-actin	GCTTGTGCGGGATATCATTTG	CCCACACCCAAAGTTCAGCCATG	GTATGTGCAAAGCCGGATTC	137	0.7593	0.0114	0.7063
COI	GCCCACTTCCACTATGTTCTC	ATAGGAGCCGTCTTTGCCATTGTTGC	CACTACACAGCACCTGAACA	108	0.5462	0.0126	0.5080
LWS-1	GAGCTGAGAAACCTTCTTT	ATGGCAGAGGAATGGGGAAAACA	GCTGTTTGTGTATGTGAATGC	116	0.7056	0.0173	0.6564
LWS-2	CAATGTGTGTCTTTGAAGGCTA	GTTGTCTCAATTTGTGGAATTGCTGGGC	CAAACTTGACATTTCCAAAGGG	123	0.8685	0.0144	0.8079
LWS-3	CAGAGGAAGGTGTGACAG	ATGGCAGAGGAATGGGGAAAACA	GCTGTTTGTGTATGTGAATGC	131	0.9694	0.0146	0.9018
LWS-R	GGGCTCTGCTTTCACATAC	CATACAAGAGATCCTTTTGAGGGACCAAACTAC	CCAAGACCAGACCATTTGTG	155	0.6412	0.0117	0.5965
Myosin HC	TCTCTGCTCACCTTCAACTTC	CCAGTTCTCGGACCCTCTGCT	CAGATCCTTGACTGTAACCTCG	136	0.5303	0.0136	0.4933
RH1	CTGGTTTGCTGGATACCTTAC	CCAGTGTGGCCTGGTACATCTTCAC	GCTTGTTCATGCAGATGTAGATC	151	0.6168	0.0131	0.574
RH2-1	ACCATCACATCTGCTCTTAA	GCTATTGAGGGATTCATGGCAACACT	AACAACCAGAGACCAGAGAG	111	0.6637	0.0139	0.6174
RH2-2	TAGGCTGTGATATGGAAGGT	GGAGGTCAAGTATCACTATGGTCTCTTGT	CCACTATGTATCTCTCAATAGCTAAG	93	0.8852	0.0258	0.8235
SWS1	GCGTTCTTCTCCAAGAGC	ACAAACAGTTCAATGCCTGCATCATGG	GTGGACACCTCAGTCTTTGA	146	0.6268	0.0164	0.5831
SWS2A	CCATCCTGCCTGTCGAAG	AGTCCTCAATAAGCAGTTTCGAACATGCATG	GTCACTGACTGAGTTGTAGAGAC	140	0.9737	0.0263	0.9015
SWS2B	TCCTGTGTCTCAAAAGCCTC	ACGTTCTCCTCAATAAGCAGTTCCGC	CACTCATCCCCAGCATCTTC	100	0.7110	0.0167	0.6614

Supplementary Table S3. PCR and sequencing primers used for LWS-1 180 Ala/Ser allele identification.

	Forward	Reverse
PCR	TGTGAAGTGCAGATCACCTAG	ACACATTCATGCATGATGCAG
Sequencing	GATCCCTTTGAAGGACCAAACT	GGACAATCATGTAGGACAGGACC
